# Case Report: Coronary plaque rupture following glucocorticoid tapering in a high-risk CAD patient with immune nephritis: mechanistic insights and clinical implications

**DOI:** 10.3389/fcvm.2025.1625491

**Published:** 2025-09-09

**Authors:** Jianxin Weng, Fushi Piao, Ruihui Lai, Wenwen Chen, Shuai Sun, Tan Xu

**Affiliations:** ^1^ Department of Cardiology, Peking University Shenzhen Hospital, Shenzhen, China; ^2^ Department of Cardiology, Shantou University Medical College, Shantou, China

**Keywords:** case report, glucocorticoid tapering, coronary plaque rupture, optical coherence tomography, immune nephritis, inflammation-driven atherosclerosis

## Abstract

**Background:**

Glucocorticoids (GCs) exhibit metabolic risks that may accelerate atherosclerosis. However, their *in vivo* effects on atherosclerotic plaques remain poorly understood. This case highlights the perilous interplay between chronic GC use and plaque vulnerability during dose reduction.

**Case summary:**

A 51-year-old male with immune nephritis, chronic kidney disease (CKD), and poorly controlled hypertension presented with unstable angina. Coronary angiography revealed multivessel disease [70% stenosis in the proximal left anterior descending artery (LAD) and 90% in the posterior descending artery]. Initial treatment included angioplasty with a drug-coated balloon in the posterior descending artery, dual antiplatelet therapy, statins, and prednisone (10 mg/day). Seven months later, after self-reducing GCs to 5 mg/day, he suffered an acute myocardial infarction due to LAD plaque rupture, confirmed by optical coherence tomography (OCT) showing fibrolipid-rich plaques, deep calcifications, and minimal lumen area (0.67 mm^2^). Emergency stenting was performed to stabilize the patient, with no recurrence at 3-month follow-up.

**Discussion:**

This case underscores the mechanistic duality of GCs. Chronic GC therapy suppresses pro-inflammatory cytokines and macrophage activity, stabilizing plaques by reducing oxidized LDL uptake. However, abrupt tapering may trigger rebound vascular inflammation, destabilizing high-risk lesions. OCT imaging proved critical in identifying vulnerable plaque morphology, emphasizing its role in guiding urgent interventions.

## Graphical Abstract

**Figure:**
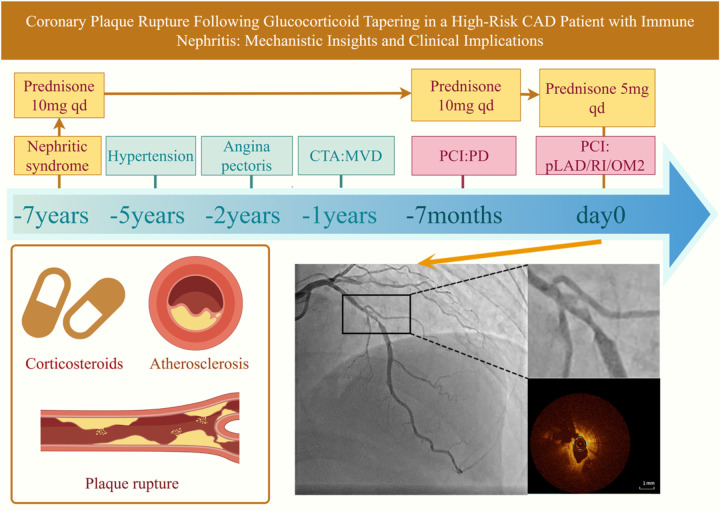

## Learning points

• This case underscores the potential role of glucocorticoids (GCs) in plaque stabilization via inflammation suppression while cautioning against abrupt withdrawal in high-risk populations.
• In autoimmune patients with coronary artery disease (CAD), GCs should be approached as a “double-edged sword”—optimizing inflammation suppression while aggressively managing metabolic risks. Advanced imaging and targeted anti-inflammatory therapies are critical tools in this high-stakes clinical scenario.

## Introduction

Coronary artery disease (CAD) often occurs in patients with multiple comorbidities, including hypertension and chronic kidney disease (CKD) (1, 2). The treatment of these patients is complex, as management must balance the risks associated with CAD, kidney disease, and medication therapy (3, 4).

Glucocorticoids (GCs), commonly used to treat immune nephritis in patients with CKD (5), have been linked to cardiovascular side effects, such as promotion of atherosclerosis and vascular wall weakening (6, 7). Although chronic systemic glucocorticoid excess is positively associated with increased atherosclerosis, emerging evidence suggests a direct causative role of glucocorticoids in the cardiovascular complications related to atherosclerotic plaque formation (6).

This case involves a 51-year-old male who initially presented with unstable angina and was later readmitted after experiencing the rupture of the left anterior descending artery (LAD), following a reduction in his glucocorticoid dose. This report highlights a rare but fatal complication of steroid withdrawal in CAD patients and reinforces the need for gradual glucocorticoid tapering in high-risk patients.

## Case presentation

A 51-year-old male was admitted to our hospital with a 2-year history of recurrent chest tightness and pain, which had worsened over the past month. He had a 5-year history of poorly controlled hypertension due to irregular use of antihypertensive medications. He also had CKD secondary to immune nephritis, managed with long-term glucocorticoid therapy (prednisone acetate, 10 mg daily). His medical history included bilateral double-J stent placement for nephrolithiasis and previous renal surgeries.

At initial presentation, the patient reported chest pain aggravated by physical activity, associated with upper limb weakness and left-sided back pain. The symptoms were partially relieved with self-administered nitroglycerin. Physical examination revealed stable vital signs [blood pressure (BP) 119/77 mmHg, heart rate 77 bpm], with no significant findings.

**Figure 1:**
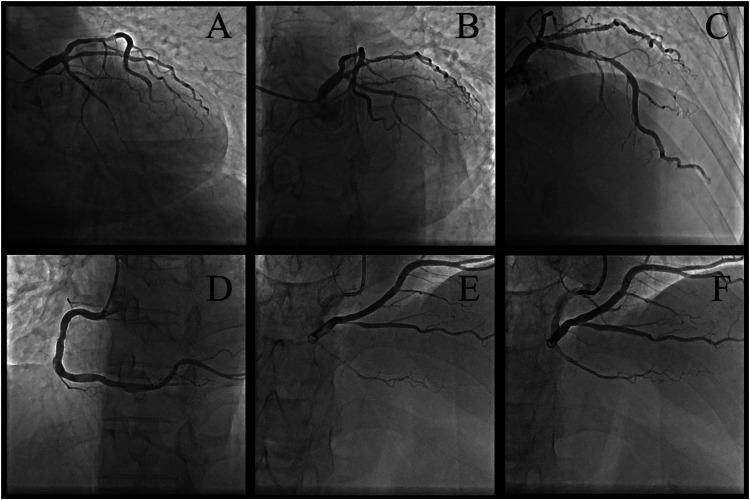
The first-time coronary angiography **(A–E)** and drug-coated balloon dilation of the PD **(F)**. Multiple projections reveal the following findings: significant coronary artery stenosis, with 70% narrowing in the proximal left anterior descending artery and 90% stenosis in the posterior descending artery. The other coronary branches showed only mild stenosis. Multiple projections for left coronary artery: **(A)** anteroposterior caudal 45°, **(B)** left anterior oblique 50°+ caudal 30°, **(C)** right anterior oblique 30°+ cranial 30°. Multiple projections for right coronary artery: **(D)** left anterior oblique 45°, **(E)** cranial 25°. **(F)** Successful coronary angioplasty with drug-coated balloon dilation of the PD.

Coronary angiography was performed on the second day after admission to clarify the etiology of chest pain. The coronary angiogram revealed significant coronary artery stenosis, with 70% narrowing in the proximal left anterior descending artery (LAD) and 90% stenosis in the posterior descending artery (PD). The other coronary branches showed only mild stenosis (Figures 1A–E). The patient underwent successful coronary angioplasty with drug-coated balloon dilation of the PD (Figure 1F).

The patient was started on appropriate medications, including statins, dual antiplatelet therapy (DAPT) (aspirin and clopidogrel), beta-blockers, and prednisone acetate (10 mg daily). His symptoms improved, and he was discharged with recommendations to continue medications, maintain blood pressure control, and implement lifestyle modifications.

## New developments and readmission

Seven months following discharge, the patient was readmitted with acute chest pain and signs of myocardial infarction. He reported increased frequency and intensity of chest pain, which did not resolve with nitroglycerin for the first time. On further inquiry, the patient disclosed that he had reduced his glucocorticoid dose from 10 to 5 mg daily under nephrologist guidance to manage his immune nephritis, as part of an attempt to minimize side effects.

**Figure 2:**
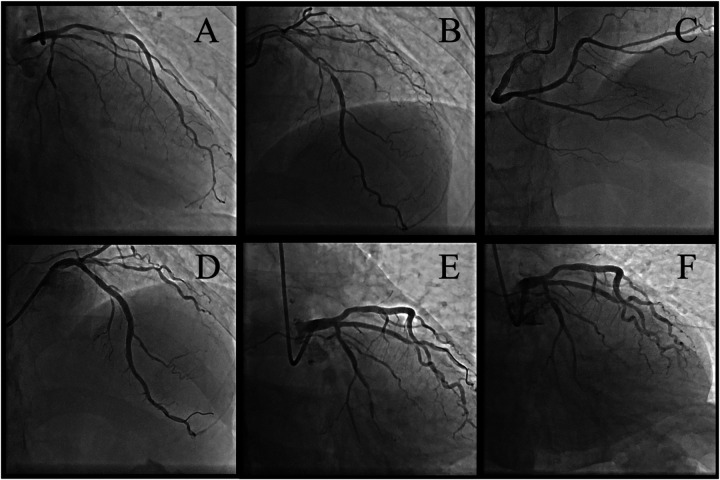
The second-time coronary angiography **(A–C)**, stent implant for left anterior descending **(D)** and ramus intermedius **(E)**, and balloon dilation for the second obtuse marginal branch **(F)**. Multiple projections **(A–C)** reveal severe multivessel coronary lesion with critical stenoses in the proximal left anterior descending (85%), ramus intermedius (80%), and second obtuse marginal branch (90%) and moderate/mild disease in second obtuse marginal branch (60%), mid-right coronary artery (50%), and left circumflex branch, posterior descending/posterior lateral (30%).

**Figure 3:**
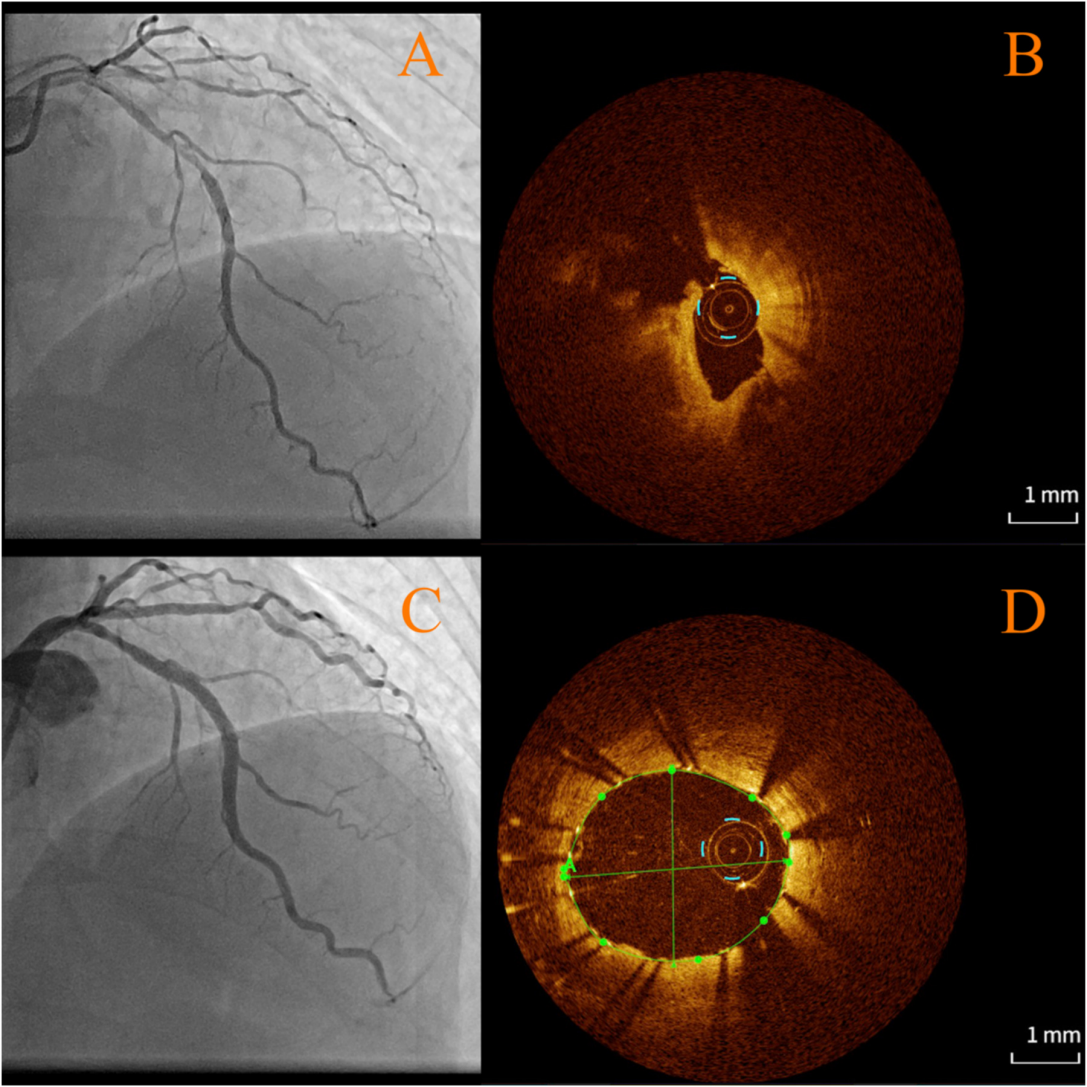
The optical coherence tomography of the left anterior descending reveals fibrolipid-rich plaques with a proximal rupture site, focal deep calcifications, a critically narrowed lumen (MLA 0.67 mm^2^), and high plaque burden **(B)**. This aligns with the angiographic progression **(A)**. **(C,D)** Angiography **(C)** and optical coherence tomography **(D)** for after stent implant in the left anterior descending.

Upon readmission, the patient was found to have a significantly elevated troponin level, suggesting a myocardial infarction. The physical examination showed no new findings; however, the ECG indicated ischemic changes (anterior leads ST segment depression >0.1 mV and T-wave inversion). Cardiac troponin T was slightly elevated (0.070 ng/ml, normal reference range <0.014 ng/ml), while cardiac troponin I was normal (0.038 ng/ml, normal reference range <0.034 ng/ml). Therefore, an urgent coronary angiogram was performed. Angiography revealed severe multivessel coronary lesion with critical stenoses in the proximal LAD (85%), ramus intermedius (RI) (80%), and second obtuse marginal branch (OM2) (90%) and moderate/mild disease in the first obtuse marginal branch (OM1) (60%), mid-right coronary artery (RCA) (50%), and left circumflex branch, posterior descending/posterior lateral (30%) (Figure 2). Compared with the coronary artery disease observed 7 months ago, significant progression has occurred in certain coronary lesions, particularly in the LAD, ramus intermedius (RI), and OM2. Furthermore, angiographic findings suggest possible plaque rupture in the LAD, based on its morphology (e.g., irregular borders, contrast staining, or ulceration). To further assess the pathology in the LAD, optical coherence tomography (OCT) was performed (Figure 3). The OCT of the LAD revealed fibrolipid-rich plaques with a proximal rupture site, focal deep calcifications, a critically narrowed lumen (MLA 0.67 mm^2^), and high plaque burden, confirming a vulnerable, unstable lesion likely driving the patient's clinical deterioration. This aligns with the angiographic progression.

Due to the rupture of the LAD, the patient underwent emergent revascularization with stenting of the ruptured segment. Meanwhile, the severe progressive lesions of RI and OM2 were also revascularized with a stent or DCB (Figures 2E,F). Following the LAD plaque rupture, DAPT was escalated to aspirin plus prasugrel. The patient reported no recurrence of chest pain or discomfort postoperatively and was discharged on optimal medical therapy with prednisone acetate (5 mg daily).

## Follow-up

At the 3-month outpatient follow-up, the patient reported no recurrence of chest tightness or angina, even during physical activity. He also demonstrated consistent adherence to his prescribed medications (dual antiplatelet therapy, statin, and prednisone acetate 5 mg daily).

## Discussion

This case is the first to report coronary plaque rupture in a patient with reduced glucocorticoid therapy. This underscores the interplay between chronic GC use and coronary plaque vulnerability. Steroid tapering in high-risk CAD patients requires gradual titration to avoid rebound potential vascular inflammation and catastrophic complications (e.g., plaque rupture). Advanced imaging (such as OCT) is critical for identifying high-risk lesions and guiding urgent intervention.

## Mechanistic duality of GCs

GCs have been reported to exert anti-inflammatory effects. However, their *in vivo* effects on atherosclerotic plaques remain poorly understood. Emerging evidence suggests that GCs inhibit pro-inflammatory cytokines (8) and stabilize vulnerable plaques by reducing macrophage activity (9) and necrotic core formation (6). Clinically relevant concentrations of glucocorticoids may shift human macrophages to a less inflammatory state, thus reducing their ability for oxidized LDL uptake (9). Glucocorticoid exposure may contribute to plaque stabilization. These findings suggest that glucocorticoids may help prevent ischemic events in patients with advanced atherosclerosis (9).

Unfortunately, glucocorticoids also have some metabolic and cardiovascular detriments (10). Chronic use of glucocorticoids exacerbates insulin resistance (11), dyslipidemia (12), and hypertension (13), accelerating atherosclerosis (14, 15). A population-based cohort study reported an increased risk of CVDs associated with glucocorticoid dose intake even at low doses (<5 mg) in six immune-mediated diseases (16). These results highlight the importance of prompt and regular monitoring of cardiovascular risk and use of primary prevention treatment at all glucocorticoid dose levels (10).

Given the mechanistic duality of GCs for the current case, it may highlight the patient's inflammation-driven atherosclerosis (e.g., likely due to immune nephritis) (17), where the anti-inflammatory effects of GCs outweighed their metabolic and cardiovascular risks. This suggests that dose reduction led to rebound inflammation, destabilizing plaques based on the plaque rupture and atherosclerosis progression in this case.

Inflammatory reactions probably increase plaque instability, possibly resulting in plaque rupture, fissuring, or erosion and setting up the substrate for the thrombotic response that causes myocardial damage or infarction (18). The Canakinumab Antiinflammatory Thrombosis Outcome Study (CANTOS) demonstrated that anti-inflammatory therapy targeting the interleukin-1β innate immunity pathway with canakinumab at a dose of 150 mg every 3 months led to a significantly lower rate of recurrent cardiovascular events than placebo, independent of lipid-level lowering (19).

In this case, an OCT evaluation was not performed at baseline due to patient's propensity. However, follow-up OCT revealed that it was indeed a lipid-rich fibroatheroma in LAD (left untreated) that subsequently ruptured and caused AMI. As the CLIMA study (Relationship Between OCT Coronary Plaque Morphology and Clinical Outcome) proved, the presence of any thin-cap fibroatheroma (TCFA) was fivefold as prevalent and similarly predictive of 5-year adverse outcomes (20).

## Clinical implications

Sustained vascular inflammation, a hallmark of high-risk atherosclerosis, impairs plaque stabilization in this phenotype (21). There is some evidence to guide glucocorticoid tapering in systemic lupus erythematosus and rheumatoid arthritis (22). However, lack of evidence to guide glucocorticoid tapering for atherosclerosis patients with autoimmune diseases, such as immune nephritis. From the case of plaque rupture, we advocate for cautious GC tapering in high-risk patients, with monitoring of inflammatory markers and plaque stability (e.g., via coronary imaging).

Some propose adjunctive therapies (e.g., advanced targeted anti-inflammatories, statins) to mitigate risks during GC withdrawal.

## Conclusion

This case underscores the delicate balance required in managing GC therapy for high-risk CAD patients with autoimmune conditions. A nuanced approach—gradual GC tapering, vigilant monitoring, and adjunctive therapies—might mitigate rebound inflammation and catastrophic events. Future research should focus on potential critical mechanisms of GC on plaque progression.

## Data Availability

The original contributions presented in the study are included in the article/Supplementary Material; further inquiries can be directed to the corresponding author/s.
